# Enabling full representation in science: the San Francisco BUILD project’s agents of change affirm science skills, belonging and community

**DOI:** 10.1186/s12919-017-0090-9

**Published:** 2017-12-04

**Authors:** Mica Estrada, Alegra Eroy-Reveles, Avi Ben-Zeev, Teaster Baird, Carmen Domingo, Cynthia A. Gómez, Kirsten Bibbins-Domingo, Audrey Parangan-Smith, Leticia Márquez-Magaña

**Affiliations:** 1 Department of Social and Behavioral Sciences, Institute for Health and Aging, University of California, San Francisco, San Francisco, CA 94118 USA; 2 Department of Chemistry and Biochemistry, San Francisco State University, San Francisco, CA 94132 USA; 3 Psychology Department, San Francisco State University, San Francisco, CA 94132 USA; 4 Department of Biology, San Francisco State University, San Francisco, CA 94132 USA; 5 Health Equity Institute, San Francisco State University, San Francisco, CA 94132 USA; 6 Division of General Medicine, University of California San Francisco, San Francisco, CA 94158 USA

## Abstract

**Background:**

The underrepresentation of minority students in the sciences constrains innovation and productivity in the U.S. The SF BUILD project mission is to remove barriers to diversity by taking a “fix the institution” approach rather than a “fix the student” one. SF BUILD is transforming education, research, training, and mentoring at San Francisco State University, a premiere public university that primarily serves undergraduates and ethnic minority students. It boasts a large number of faculty members from underrepresented groups (URGs), including many of the project leaders. These leaders collaborate with faculty at the University of California San Francisco (UCSF), a world-class medical research institution, to implement SF BUILD.

**Key highlights:**

Together, the campus partners are committed to creating intellectually safe and affirming environments grounded in the Signaling Affirmation for Equity (SAFE) model, which is based on robust psychosocial evidence on stereotype threat and its consequences. The SAFE model dictates a multilevel approach to increasing intent to pursue a biomedical career, persistence in STEM fields, and productivity (e.g. publications, presentations, and grants) by implementing transformative activities at the institutional, faculty, and student levels. These activities (1) increase knowledge of the stereotype threat phenomenon; (2) affirm communal and altruistic goals of students and faculty to “give back” to their communities in classrooms and research activities; and (3) establish communities of students, faculty and administrators as “agents of change.” Agents of change are persons committed to establishing and maintaining SAFE environments. In this way, SF BUILD advances the national capacity to address biomedical questions relevant to communities of color by enabling full representation in science.

**Implications:**

This chapter describes the theoretical and historical context that drive the activities, research and evaluation of the SF BUILD project, and highlights attributes that other institutions can use for institutional change. While this paper is grounded in psychosocial theory, it also provides practical solutions for broadening participation.

## Context and building on opportunity

**Fig. 1:**
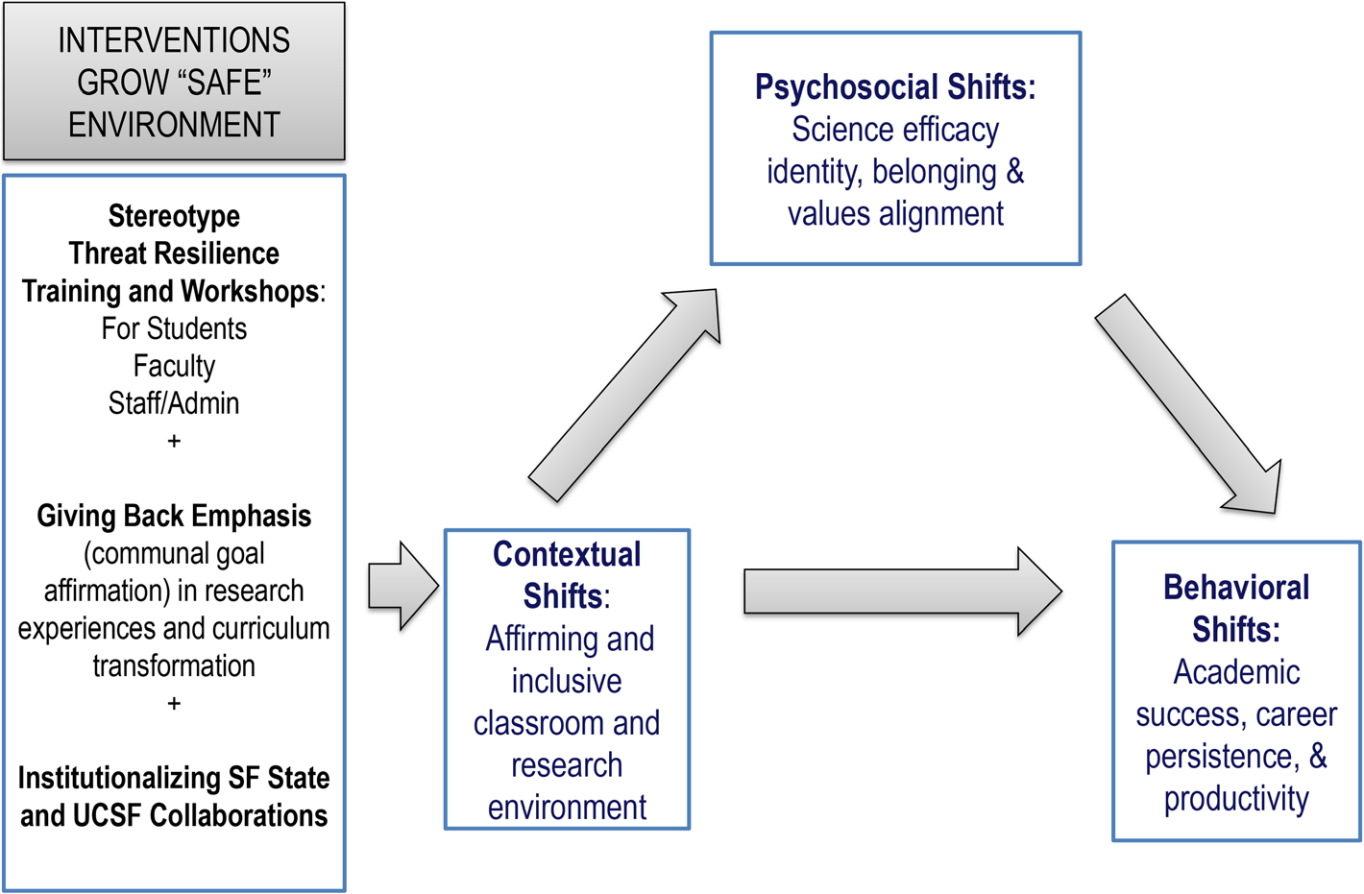
Signaling Affirmation for Equity (SAFE) model. This model describes project activities as contributing to building a more affirming and inclusive context, which then leads to greater academic success, persistence and productivity. Consistent with a robust literature on persistence, this later relationship is mediated by science efficacy, identity, belonging and value alignment. This model shows the logic that building a SAFE environment ultimately results in fuller representation of students and faculty in the biomedical research workforce

The need for the national scientific enterprise to better include and address the needs of historically underrepresented students and the communities from which they come is a national mandate [1, 2]. San Francisco Building Infrastructure Leading to Diversity project (SF BUILD: http://sfbuild.sfsu.edu) aims to fulfill this mandate through supporting a suite of programs and activities that create intellectually safe and affirming environments grounded in the Signaling Affirmation for Equity (SAFE) model (see Fig. 1). The SAFE model is based on robust psychosocial evidence on stereotype threat and was developed for this project. Stereotype threat is a social contextual phenomenon which occurs when people from stigmatized groups experience concern about confirming negative group stereotypes [3]. The experience of stereotype threat is, in turn, a predictor of URGs’ early exit from science. With this model as a guide, SF BUILD programs and activities increase awareness of stereotype threat at all campus levels: institutional, faculty, and student. SF BUILD also works to guard against the negative impacts of stereotype threat by providing opportunities for URG faculty and students to “give back” to the larger community as scientists.

SF BUILD is responding to the need for scholarly approaches that link student persistence outcomes to the academic institutional contexts that drive them. The project’s goals are to broaden participation, create student success, and promote social equity, while decreasing the inequities in education that can perpetuate white privilege [4]. Thus, instead of taking the traditional “fix the student” approach, SF BUILD focuses on “fixing the institution” by cultivating a community in which informed *agents of change* contribute towards broader participation in science. Focus on institutional change is not common among diversity programs that historically have been grounded in a student-deficit model [5]. Researchers of higher education also broadly hold this view. In fact, scholars lament that few higher education researchers engage in the “study of institutional-level contexts where diversity dynamics play out” [6].

Meanwhile, layered beneath the institutional, faculty and student level activities is a research program to assess when and why this approach works or does not work. Importantly, research and SF BUILD project activities are not separate from one another, but work to inform each other, utilizing an action research approach (see [7] for fuller description of action research). While the success of project activities is largely measured by assessing student and faculty outcomes (i.e., individual level measures), SF BUILD uses this information to inform and initiate institutional changes over the long-term. The process of documenting the methods and outcomes of transformation, while iteratively conducting trainings and creating opportunities to connect biomedical science to student values, is central to the SF BUILD process. In this way, the project is actively creating a biomedical research workforce that more accurately reflects the diversity of our country.

### SF BUILD context

Effective institutional change management strategies require understanding of the current cultural context into which agents of change are to emerge and act. For SF BUILD, the contexts are the campuses of San Francisco State University (SF State) and its partnership with the University of California San Francisco (UCSF).

In a 1995 keynote address to SF State graduates, then First Lady Hillary Rodham Clinton honored the university with the following words, “SF State is a great public university. A university that takes the education of all people seriously*.*” This national recognition of SF State’s commitment to include all people in higher education reflects the university’s historical legacy and its current demographics. SF State is a predominantly undergraduate institution that has attained national prominence for scientific teaching, research training, and preparation of underrepresented students for biomedical research careers. SF State teaches and trains a student population of 27,000, with 89% of the students being undergraduates. Nearly 35% of the undergraduates are first generation university students and 2.8% of the students receive services from the Disability Programs and Resource Center. Of those declaring their ethnicity in the Fall 2014 semester, ethnic minorities comprised 68.2% of the undergraduates and 42.7% of the graduate students. In total, 16,014 of the enrolled students are ethnic minorities. Sixty-four percent of these are from the four federally designated UMGs (African American, Latino, Native American, Pacific Islander, and More than one Ethnicity – including one of a UMG). Unfortunately, when retention and graduation rates are disaggregated by race/ethnicity, it becomes abundantly clear that SF State mirrors the United States in that the different racial/ethnic groups have different outcomes of degree attainment [8]. Institutional deficits that can perpetuate inequitable degree attainment are a target of SF BUILD innovations [4]. SF BUILD capitalizes on the rich ethnic diversity at SF State for addressing these systemic deficits. In fact, SF State is the most ethnically diverse university in the country when compared to other large universities [9].

### Agents of change at SF State

In addition to understanding the context, institutional levels of change require current institutional structures to be “unfrozen,” or open to movement, to foster institutional transformation [10, 11]. Unfreezing happens when there is destabilization or a shared perception that the status quo is no longer resulting in the achievement of institutional aims. SF BUILD benefits from being in a context that has a history of being “unfrozen” and reshaped. SF State was founded in 1899 as a university for preparing future teachers, and, one year after its establishment, the school’s teaching methods were defined as “radical.” In fact, a quote on SF State’s website referencing historical events that transformed the academy proudly states: “We’ve been shaking things up ever since.” This sentiment is best illustrated by the student-led strike of 1968-69. The protest was ignited by several factors, including the numerical underrepresentation of students of color in the late 60’s, the virtual lack of faculty of color, and the absence of curriculum that included the experiences of people of color in the United States. The five-month strike was led by a relatively small community of students of color that were supported by faculty and staff, as well as members of the local community. The strike made national news and sparked similar actions across the country that were chronicled in the documentary film “Agents of Change” (http://www.agentsofchangefilm.com/). The actions of the strike leaders and supporters (i.e., the agents of change) resulted in the creation of the only College of Ethnic Studies in the nation and an institutional commitment to inclusion of people of color in SF State’s teaching practices, engagement with community, and research. This commitment is particularly true for the social sciences, but efforts to include the experiences of communities of color in both the curriculum and research of the biomedical sciences at SF State lag behind. SF BUILD activities are picking up the pace of these inclusion efforts at SF State.

### History of SF State and UCSF collaboration in training

In 1992, SF State initiated its first training program for students in collaboration with UCSF in order to broaden participation in research. UCSF, a research-intensive university, is consistently ranked as one of the top five universities receiving the most funding from the National Institutes of Health (NIH) [12]. As such, UCSF has stepped up to the national mandate, voiced by NIH leaders [2], for promoting diversity by working with SF State to address institutional deficits that maintain underrepresentation of particular groups in science. This ongoing collaboration with UCSF exposes SF State students pursuing bachelor and master’s degrees to doctoral degree training that is not available at SF State. The cross-institutional partnership also provides exposure and opportunities for faculty research collaboration that benefits both institutions. The NIH has supported these types of collaborations over the years. Since 1993, it has funded the Bridges to the Future program, as well as funding many other student and faculty training programs that partner SF State and UCSF. As a result, UCSF has become a prominent destination campus for SF State graduates. The previously existing partnership between SF State and UCSF consists of both formal and informal inclusion of SF State students in summer programs and year-round research experiences. These have resulted in a dramatic increase in the number of students from SF State who enter and complete PhD programs [13]. Until the funding of SF BUILD, however, a theoretical model has not guided these joint efforts to train students and promote faculty collaboration, nor have the efforts been strategically executed and evaluated. Formalizing these efforts through SF BUILD has resulted in an increase in the number and areas of expertise of UCSF faculty collaborating with SF State faculty and training SF State students.

## SF BUILD extends a legacy of inclusion using a theory driven approach

### The signaling affirmation for equity (SAFE) model

The SF BUILD approach is grounded in social science theory regarding stereotype threat and emerging work on the value of individual and group affirmations to mitigate and overcome psychosocial barriers to success in science. There is strong evidence that, in addition to having the skills to pursue a science career, the social experience of a student is even more predictive of their persistence in a STEM (Science, Technology, Engineering & Math) field [10]. Research on stereotype threat shows that negative environmental cues can signal that underrepresented minority students “do not belong,” resulting in detrimental effects on the achievement and motivation of these students (e.g., [14]). For example, underrepresentation of women and some ethnic minorities in STEM environments can be powerful triggers of stereotype threat [3, 15]. As a result, individuals subjected to the social psychological context of ST perform below their abilities, culminating in heightened attrition and a perpetuation of underrepresentation [3, 14, 16, 17]. The constant experience of stereotype threat can result in poor academic performance, a reduced sense of belonging, and lack of professional identity [18–20]. In addition, a reduced sense of belonging in the scientific community can impede the development of a strong identity as a legitimate scientist [21], which has been repeatedly shown to predict URG persistence in the biomedical research workforce [22, 23].

Building on the stereotype threat literature, the SF BUILD team developed the Signaling Affirmation for Equity (SAFE) model by integrating findings from several lines of research on the documented psychosocial barriers to success in science (see Fig. 1). The SAFE model dictates a multilevel approach to increasing intention to pursue a biomedical career, persistence in STEM fields, and productivity (e.g. publications, presentations, and grants) by implementing transformative activities (see below) at the institutional, faculty, and student levels. Found in Table 1 these activities (1) increase knowledge of the stereotype threat phenomenon, (2) affirm communal and altruistic goals of students and faculty to “give back” to their communities using science, and (3) establish communities of change (aka, agents of change). Two studies at SF State in addition to previous social science work informed the SAFE model [24, 25]. The current articulation of the SAFE model for the SF BUILD project (as shown in Fig. 1) has been instrumental in its implementation and will continue to be critical in allowing the project to reach its goals.

**Table 1:** Key Features of SF BUILD Activities

Conducting Stereotype Threat (ST) Resilience Workshops & Trainings	
● ST is explained and participants identify how it occurs in their own lives. ● ST impacts are described utilizing current research and examples are provided of how to turn ST into a challenge. ● Strategies are developed and shared on how to reduce ST in the classroom and research environment.	
Creating a “Giving Back” Emphasis in Biomedical Sciences	
● Opportunities are given to share knowledge with others (through mentorship, instruction, collaboration). ● Personal and lived experiences are welcomed in classrooms and research environments and inform research questions relevant to diverse communities. ● There is intentional building of institutional commitment to support and value community-engaged research.	
Institutionalizing SF State and UCSF Collaborations	
● Providing programs, space and resources for cross-institutional research to occur in SAFE environment (e.g., SF BUIILD Scholars program and SOUL^a^). ● Supporting classroom shifts that reduce ST and increase “giving back” curriculum. ● Hosting ST Resilience workshops, mentor training, and cross-institutional working groups to support SF State and UCSF faculty collaborations in grant making, scientific writing and creating affirming environments for professional development.	

^a^Social Innovations and Urban Opportunities Lab (SOUL) is the first joint research facility to formally partner SF State and UCSF investigators. It was created as a result of BUILD funding

## Affirming student engagement in biomedical research careers

### Primary aim and signature activities

Faculty, staff, and administrators working directly with students design activities and opportunities for students that provide education about stereotype threat and how to combat it; enable investigation of biomedical research questions relevant to local communities so as to make clear that they can “give back” to their communities of origin; and develop skills to teach or mentor other biomedical students to give back more immediately.

### Pathways for student engagement

In the signature SF BUILD scholars program, SF BUILD selects 11-12 students annually to receive financial support and training through the SF BUILD scholars program. Students who are two years from graduation at SF State are chosen from among those enrolled in biomedical majors, 37% of which come from underrepresented groups in the sciences (African American, Latino, Native American, Pacific Islander, and multi-ethnic persons with one parent being from an URG). Scholars delve into learning about stereotype threat and actively engage in research training activities that affirm their capacity to “give back” and reach communal goals via a 10-week summer program. During the program, the Scholars rotate through four research labs that introduce them to biomedical investigation at the molecular, individual, societal, and population levels (three at SF State and one at UCSF). The following academic year, SF BUILD Scholars select a faculty mentor with whom to work, either at SF State or UCSF. The majority of these faculty members are themselves from URGs. During rotations, scholars also participate in a suite of planned training, group activities, and career development activities.

The SF BUILD Scholars also play an important role in extending the program’s reach through their student networks – in this way becoming agents of change on campus. In fact, this role is part of our retention strategy that affirms the communal goals of the scholars by providing them with structured opportunities to use science in their communities and describe their work to others. This is also accomplished through their engagement in near-peer mentoring activities with Metro Science students that increase self-efficacy, sense of belonging, and science identity [25, 26]. The Metro Science program is a component of the Metro College Success Program designed to support first-generation, low-income and/or historically underrepresented students to succeed at SF State. The near-peer mentoring involves Scholars learning how to support slightly more junior students who are pursuing similar career objectives. As such, they become representatives of their field to other students and are able to immediately give back to others, becoming agents of change.

In addition to the SF BUILD scholar program, SF BUILD has worked to support and expand the Supplemental Instruction (SI) program, an evidenced-based academic support intervention. The SI program provides specialized training to student instructors for STEM courses. Aimed at improving academic success, each SI course is taken concurrently with gateway classes in math, chemistry, physics and biology [27, 28]. Senior undergraduate students facilitate peer learning between undergraduates in a workshop setting that is in addition to, and supportive of, the typical entry-level science course instruction. Participation in SI is correlated with higher mean grades along with higher retention and graduation rates [27]. SF BUILD faculty leaders provide training for SI instructors in stereotype threat theory and provide SI course instructors with tools to combat this threat. They also teach their student instructors how to address the students’ altruistic and communal goal values. This training deepens student knowledge of key psychosocial phenomena, as well as strengthens their ability to instruct others in their field. This also serves to build their own efficacy and identity within the field.

Last, the SF BUILD project has supported several course-based interventions that are informed by social science studies on motivation and persistence. For example, one intervention was to have STEM students engage in thinking about and finding ways to respond to stereotype threat. This was a onetime online intervention. Another study, based on utility value theory, connected course content to personal values. The long-term impact on retention and persistence for students exposed to these larger scale interventions will be determined in summative evaluations of the SF BUILD project, but interventions that result in short-term increases in motivation and persistence will inform ongoing curricular change.

### Connection to theory

All students participating in SF BUILD activities have different degrees of experience with the SAFE model. The SF BUILD Scholars have the most comprehensive experience. Upon admission to the training program, they engage in research experiences and cohort building activities that encourage them to think about and explore the ideas of stereotype threat. They are also shown the various ways they can use their scientific training to address biomedical questions relevant to URG communities. Unlike the SF BUILD Scholars and SI instructors, students in SI classes do not directly explore this theory. To measure the impact of SF BUILD student activities, we are measuring students’ science efficacy, identity, sense of belonging and awareness about stereotype threat. In classroom based activities, we are also measuring when classroom environments, in which instructors are working to reduce stereotype threat, increase connections between course content, and affirming “giving back” communal goals. Overall, SF BUILD activities, which seek to create SAFE environments, have directly impacted hundreds of students pursuing biomedical research related degrees and many more through SF BUILD faculty training and institutional change activities described later in this chapter.

## Cultivating faculty agents of change

### Primary aim and signature activities

The overall aim of SF BUILD faculty activities is to provide professional development opportunities for faculty to become agents of change and shift campus culture towards a more affirming environment for all students, particularly in biomedical science classes. To achieve this, SF BUILD delivers workshops for instructors and research advisors to increase knowledge of the impact of stereotype threat in the classroom and identify ways to decrease threat for students and other faculty. More than 200 individuals have participated in these workshops to date. Secondly, a smaller group of faculty are participating in faculty learning communities where they learn about psychosocial and science education literature and then develop theoretically grounded classroom and research training interventions. These interventions are aimed at promoting a SAFE environment and focus on affirming student “giving back” communal and altruistic goals.

### Pathways for faculty engagement

SF BUILD has engaged the majority of faculty through providing trainings to help faculty create teaching and research environments for students that are affirming, productive, and intellectually safe spaces. Specifically, faculty members at SF State and UCSF have attended workshops to learn about stereotype threat theory, how to implement strategies to combat this threat, and how to teach in ways that support student altruistic and communal goal values. For example, as described in Table 1, during SF BUILD led workshops, faculty are encouraged to reframe potentially threatening situations or activities, and identify mindsets and resources to convert the threat into a challenge [29]. At SF State, these trainings have occurred during new faculty orientation sessions, annual faculty retreats, and during departmental meetings. SF BUILD has also developed training workshops on effective mentoring to improve the experience of students in research labs who may be numerically underrepresented with regard to gender and/or ethnicity. Likewise, UCSF faculty and post-doctoral fellows (future faculty) engage in activities that teach them how to both recognize the triggers of threat, and generate ideas for combatting it. They are then guided in developing practical tools to combat triggers of threat in their own research environments and mentoring relationships.

**Fig. 2:**
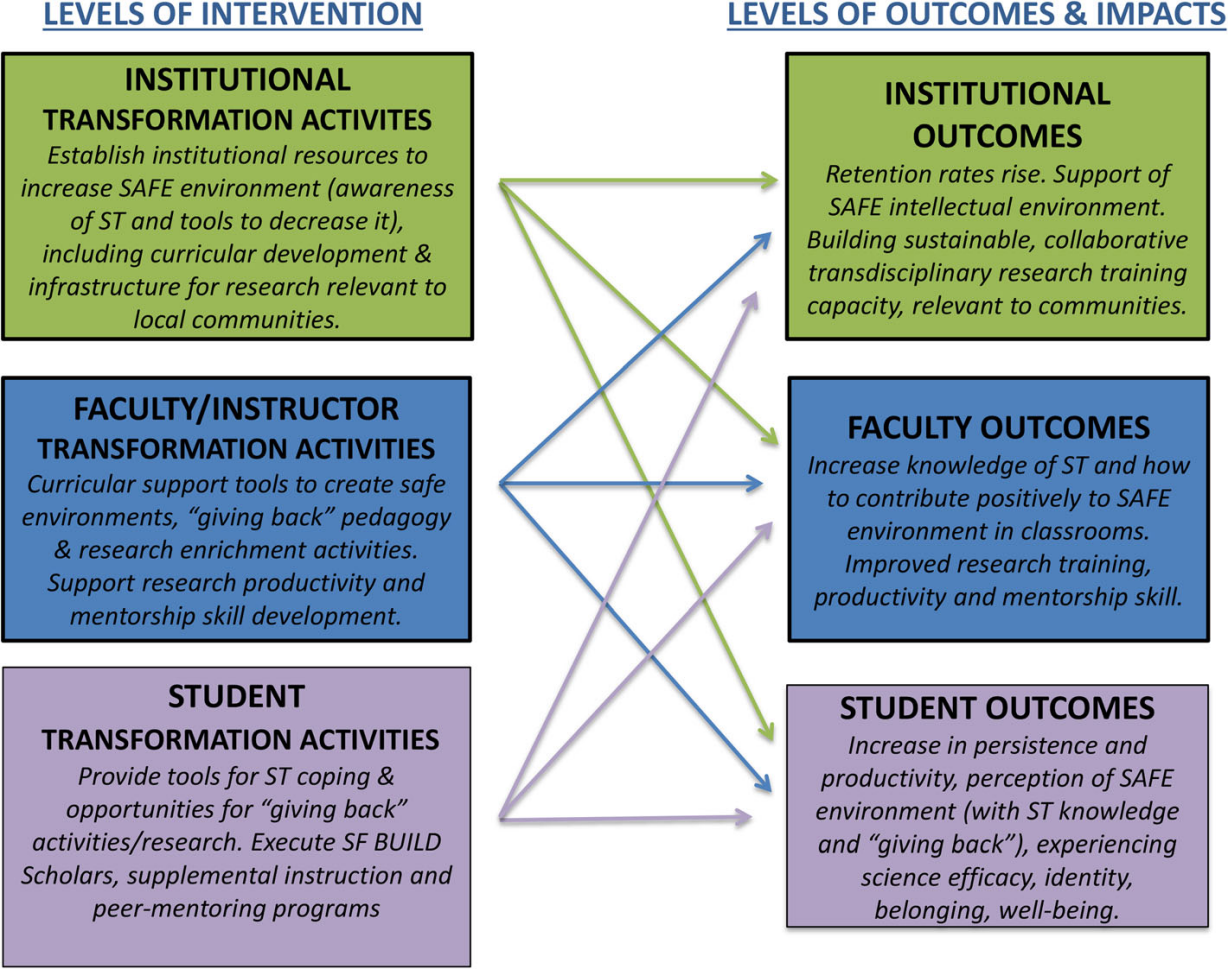
Illustration of levels of interventions and predicted outcomes of SF BUILD project

For faculty that want to more deeply explore the impact of stereotype threat on the student experience, the Faculty Agents of Change (FAoC) Initiative [30] was established to allow for more in depth scholarly discussion of relevant articles documenting systemic barriers and facilitators of success for underrepresented students in science. These communities adopt the SAFE model as a compelling philosophy that is used to improve teaching and enhance curriculum (see Table 2). Notably, these communities include basic and social scientists, as well as science educators. The trust that exists in these faculty learning groups (@10 people each) promotes the sharing and critiquing of ideas for classroom-based modifications. In addition to benefitting from a supportive community, faculty in these communities of transformation also benefit from significant resources, including release from coursework and seed funding for project implementation. For example, faculty members from various departments are working to develop and evaluate the efficacy of “Social Justice Pedagogy” to be used in various quantitative classes focused on research methods. When integrated into the SF State curriculum, this new way of teaching is expected to yield curricular transformation at the institutional level (see Fig. 2). This transformation will result in the affirming of the communal goals of both students and faculty. This, in turn, mirrors the Association of American Universities Network Framework, highlighting system-level elements required to create institutional change [31]. At the institutional transformation level, SF BUILD Leadership is working to ensure that FAoC efforts are valued in the retention, tenure, and promotion process. This allows faculty to fully engage in the activities that are expected to shift the culture of science in teaching and research. The FAoC initiative also includes developing, implementing, evaluating and disseminating trainings and tools (e.g., short class modules for linking class content to student communal values) that are found to be effective for student persistence for other departments and institutions.

**Table 2:** Key Features of Agents of Change

A SF BUILD Agent of Change…	
● commits to promoting a SAFE environment in the classroom, research environment, and among peer groups. ● engages in critical reflection of own teaching, research mentoring or peer-mentoring practices that may influence student self- efficacy, science identity, and sense of belonging. ● implements interventions that institutionalize attributes of a SAFE environment.

### Connection to theory

SF BUILD expects that training SF State and UCSF faculty to develop and use tools for combating stereotype threat and promote communal goals will shift classroom and research training cultures towards more inclusive and affirming environments. Initial data shows that this type of environment increases student science efficacy, identity, and sense of belonging as diagrammed in the SAFE model (Fig. 1), resulting in greater persistence.

## Creating institutional change

### Primary aim and significant activities

SF BUILD institutional change activities aim to foster connections between SF State and UCSF, as well as garner commitment from these institutions in the form of space, resources, and policy commitments. A primary mechanism for achieving the former has been to provide opportunities for cross-institutional collaborations on grant proposals and projects that investigate biomedical research topics relevant to local communities. To further solidify SF BUILD objectives, institutional leaders have supported increasing knowledge of stereotype threat through institutionalizing workshops and orientations (e.g., workshops at the annual faculty research retreat and new faculty orientation). Elements of these workshops will be made available to other institutions wishing to create a more affirming environment by dissemination of toolkits for promoting resiliency to stereotype threat. These toolkits will be housed both at SF State and UCSF and plans are to provide technical assistance for their adaptation and use.

### Approach to fostering institutional change

Despite the need for change, approximately 70% of institutional change efforts fail [32]. With this in mind, SF BUILD is utilizing change management theories from the organizational behavior field to guide its efforts. These theories have been developed in business settings and provide frameworks for structured processes that ensure that changes are systematically and efficiently implemented for lasting benefits. The emergent approach emphasizes that change is not a linear process, but a continuous, open-ended process of adaptation to changing circumstances and conditions. This approach is particularly well-suited to institutions of higher education because change is attained through a process of continuous learning [33, 34]. The approach focuses on the processes and activities that lead to greater understanding of the complexity of the relevant issues, the identification of the range of available options, and the gathering of information on short-term outcomes. These results guide ongoing work to create sustainable institutional change.

A critical step of an emergent change management approach is the creation of a shared vision [35]. For SF BUILD this was accomplished via development of the SAFE model (see Fig. 1). To promote and solidify a shared vision, all SF BUILD materials and communication strategies maintain key messaging grounded in our SAFE model. The communication of a consistent vision empowers institutional leaders -- including administrators, staff and faculty -- to better understand, communicate and act as effective agents of change on campus. Another method of maintaining a shared vision is to celebrate success, establishing new perceptions of normative institutional behavior. This type of momentum for change is further propelled by the creation and consistent highlighting of short-term wins and regular broadcast of successes [35].

To implement and sustain ongoing change, the gathering of data and anchoring change efforts to institutional culture is recommended [35]. Our program of evaluation and social science research meets the first criterion. The framing of SF BUILD efforts as extending the legacy of the 1968 strike at SF State grounds it in an institutional “agents of change” culture.

### Mechanisms for deepening ties between partner institutions

In addition to sharing communication strategies, SF State and UCSF have joined together to create cross-institutional research and research training opportunities to enhance NIH-funded research at SF State and leverage existing efforts at UCSF. Two existing NIH-funded center grants that include educational cores devoted to increasing underrepresented minority researchers have the specific goals of promoting collaborations between UCSF and SF State, and to provide both didactic and experiential research training for SF State students. For example, the NIH funded **C**enter for **H**ealth **A**nd **R**isk in **M**inority youth and adults (**CHARM**) and the **Bring It Down Study (BID)** create forums for UCSF faculty to mentor SF State URG faculty. These forums enhance faculty career development and promote additional collaborations between the partner institutions. SF BUILD has also initiated: 1) *quarterly SF BUILD Dialogues* that provide opportunities for faculty across the institutions to network and discuss shared research interests with expert speakers; 2) *writing groups and retreats* that provide technical assistance for preparing manuscripts and grant applications and stimulate collaborations; 3) *mini-grant* opportunities that provide seed money for faculty to engage in innovation and research; and 4) *enhanced support of new joint research* infrastructure and training opportunities (e.g., a core facility that supports place-based research relevant to local communities). Overall, SF BUILD has carefully directed resources to optimize institutionalizing opportunities for SF State and UCSF faculty to be actively engaged in building research partnerships. Faculty report that these activities contribute to broadening productivity and reach for both faculty and trainees across the two institutions.

Institutionalization efforts have not only focused on creating strategic partnerships, but include mechanisms for responding rapidly to moments of change. Case in point, during the first months of the grant, UCSF students staged a ‘die-in’ to call attention to issues of race. Over 200 graduate and professional students at UCSF protested the loss of black lives by lying down in the center of campus in mock death [36, 37]. Student protestors were dressed in white coats, a sign indicating that the ‘die-in’ was connected to similar actions at 50 campuses across the country organized via social media (#whitecoats4blacklives). The garnered national attention sparked UCSF administrators to restructure the annual School of Medicine retreat to focus on the theme, “Race Matters,” and SF BUILD was the topic of an opening session. In the year since the ‘die-in’, the SF State Provost, Sue V. Rosser, PhD, delivered the commencement speech for the UCSF Graduate School wherein she described the importance of inclusion to the practice of science. This message, as it relates to the practice of both science and medicine, has also been advanced by a variety of high-level UCSF administrators. Furthermore, UCSF investigators and SF BUILD team members have documented the importance of inclusive practices to increase the rigor and impact of biomedical studies [1]. Much like the SF State strike that took place nearly fifty years earlier, student-led action unfroze the status quo and created a context for change. This enabled SF BUILD to deepen the ties between the partner institutions that continue to provide opportunities for collaboration on issues of diversity at both institutions.

### Connection to theory

SF BUILD seeks to institutionalize a SAFE environment that is characterized by less experiences of stereotype threat, opportunities to give back in research and classrooms, and greater collaboration between partner institutions. The model presented in Fig. 1 shows that by shifting the practices and culture of the institution toward a more SAFE environment, increased retention, persistence and productivity are ultimately predicted. We aspire to collect data showing positive gains in all of these areas so as to provide evidence of how institutional transformation is an effective and sustainable approach to addressing the urgent need to broaden participation of all groups in biomedical research.

## Gathering evidence to meet the national need for diversity in biomedical research

### Evaluation aims

SF BUILD achieves evaluation through program assessment and social science research. This is to say that, in addition to reaching benchmarks that indicate transformation occurs at all levels (institutional, faculty, and student), we are also interested in “why” these transformations do or do not occur. Importantly, our evaluation measures the efficacy of interventions (e.g., signature activities described previously in this chapter) that target transformation at the three levels. For this reason, the design of our evaluation is complex and multifaceted. These features are illustrated in Fig. 2 which shows that the level of the SF BUILD project signature activities can result in outcomes at all of those same levels. For instance, providing faculty with practical tools for creating SAFE environments for students may result in institutionalizing the use of these tools, faculty satisfaction, and differential learning for students. We therefore aim to measure these kinds of outcomes utilizing student and faculty self-reports and institutional data.

## Evaluation method

A great strength of our assessment approach is that there are a variety of quasi-experimental research projects imbedded in it. Whenever possible, we are collecting data from participants engaged in SF BUILD activities and a comparison group. Specifically, to get a better understanding of “why” signature activities have the impacts they do, we are collecting measurements of psychosocial variables (e.g., perceptions of stereotype threat, micro affirmations, efficacy, science identity, values, and belonging). The investigation of these psychosocial variables is expected to advance the science needed to meet the critical need for diversity [2]. In particular, our evaluation efforts are expected to identify moderators and mediators for persistence and success in biomedical disciplines for both majority and underrepresented students. What follows are descriptions of some of the significant quasi-experimental efforts embedded in our research and evaluation program.

Study 1: SF BUILD Scholar Stereotype Threat Study. This study seeks to longitudinally track the impact of participating in the SF BUILD scholar program on retention in biomedical majors, persistence in biomedical research careers, and psychosocial factors that have been shown to predict retention and persistence. By the end of the five-year funding period, SF BUILD scholars are expected to show a decrease in perceptions of stereotype threat as well as an increase in science identity, sense of belonging in science, self efficacy, and preparedness for and desire to pursue graduate studies in science. For each yearly cohort of 11-12 SF BUILD Scholars (experimental group) there are two control groups: NIH MARC scholars and non-NIH affiliated SF State scholars. The latter are matched with SF BUILD scholars on important variables, such as ethnicity, gender, major, GPA, etc. In addition, qualitative data will be collected via one-on-one interviews. The goal of these interviews, in general, is to understand more deeply and in a nuanced way the psychosocial factors that emerge from students’ training on stereotype threat and strategies to overcome it, and as a result of their research and academic experiences in SF BUILD.

Study 2: Speaking Truth to Empower (STEP). Since 2015, Avi Ben-Zeev has been heading a team effort to generate and test a novel intervention, *Speaking Truth to Empower* (STEP). The intervention, which is in line with the SAFE model, is designed to combat stereotype threat in STEM (Ben-Zeev et al., *in progress*). STEP takes a ‘knowledge as power’ approach, which holds that it is both ethical and effective to be upfront with URGs about what might adversely impact them (also see [38]). STEP consists of a *knowledge component*, which entails teaching students about stereotype threat and an *actionable component*, which involves asking students to generate an example about a stereotype threat situation they have experienced. They are then asked to imagine how they might react to the same situation in the future or how they might help a peer cope with a similar situation someday. A study conducted with 670 participants enrolled in STEM majors at SF State has shown promising results: STEP not only seemed to protect intellectual performance and sense of belonging under stereotype threat, but importantly, it appeared to reduce URGs’ concerns with stereotype-based evaluations, which have been theorized and documented to be integral and causal to achievement [3].

Study 3: Illuminating Pathways Study. We are collecting data from students in biology, chemistry, engineering and mathematics regarding their experiences at SF State, psychological variables, and intentions to persist in STEM fields. We are coupling this with outcome variables such as GPA and course persistence acquired through the institutional research office. In year two of the project, we collected data from > 700 students. Our intention is to collect similar data annually as a measure of institutional shifts and departmental shifts. The level of administration and faculty support is enormous. In exchange for their support, we are able to provide faculty and administrators with data about their students that can inform their approach to teaching and administration going forward. In this way, our evaluation efforts are firmly placed within the emergent change management approach that dictates the gathering of data to implement and sustain ongoing change within an institution.

### National level study of diversity consortium

In addition to local studies, the SF BUILD team is collaborating with the Coordination and Evaluation Center (CEC) to track engagement and outcomes of students and faculty engaged in SF BUILD activities [39].

## Expected impact of SF BUILD on biomedical research

The workforce is limited by the fact that non-minority scientists are less likely than their minority counterparts to pursue questions specific to communities of color [40], diminishing access to the benefits accrued. This is particularly noticeable in the realm of biomedical research. Therefore, broadening the participation of all groups in the scientific workforce will better ensure that federally-funded research is rigorous and meaningfully addresses issues relevant to all tax-paying populations [1]. Through SF BUILD, equity in biomedical research participation is beneficial and will be more effectively achieved by supporting student and faculty to be agents of change, promoting a SAFE environment at other institutions touched by their work. Agents of change are critical to strategic science efforts [41] and have the potential to impact the national dialogue on this topic. The SF BUILD activities that promote “giving back,” community-engaged research approaches are arguably the best way to achieve the NIH mission of “Turning Discovery into Health” because of their transformative and translational nature [42]. SF BUILD contributes to efforts to attain these translational outcomes for all U.S. populations by eliciting institutional transformation at a nationally recognized public university that serves large numbers of students who are underrepresented in science (SF State), and a top, NIH-funded institution (UCSF). Together, the work of these partner institutions show that institutional change is possible, and that SF BUILD’s approach is an effective and sustainable approach to addressing the urgent need to broaden participation of all groups in biomedical research.
